# Enhanced Bone Healing in Critical-Sized Rabbit Femoral Defects: Impact of Helical and Alternate Scaffold Architectures

**DOI:** 10.3390/polym16091243

**Published:** 2024-04-29

**Authors:** Iván Alonso-Fernández, Håvard Jostein Haugen, Liebert Parreiras Nogueira, Miriam López-Álvarez, Pío González, Mónica López-Peña, Antonio González-Cantalapiedra, Fernando Muñoz-Guzón

**Affiliations:** 1 Anatomy, Animal Production and Veterinary Clinical Sciences Department, Veterinary Faculty, Universidade de Santiago de Compostela, Campus Universitario s/n, 27002 Lugo, Spain; monica.lopez@usc.es (M.L.-P.); antonio.cantalapiedra@usc.es (A.G.-C.); fernandom.munoz@usc.es (F.M.-G.); 2 Department of Biomaterials, Institute of Clinical Dentistry, Faculty of Dentistry, University of Oslo, 0317 Oslo, Norway; h.j.haugen@odont.uio.no (H.J.H.); l.p.nogueira@odont.uio.no (L.P.N.); 3 Centro de Investigación en Tecnologías, Energía y Procesos Industriales (CINTECX), Universidade de Vigo, Grupo de Novos Materiais, 36310 Vigo, Spain; miriammsd@uvigo.gal (M.L.-Á.); pglez@uvigo.gal (P.G.); 4 Galicia Sur Health Research Institute (IIS Galicia Sur), SERGAS-UVIGO, 36213 Vigo, Spain

**Keywords:** polylactic acid, bioceramic, 3D-printing technology, composite scaffolds, scaffold architecture, bone regeneration

## Abstract

This study investigates the effect of scaffold architecture on bone regeneration, focusing on 3D-printed polylactic acid–bioceramic calcium phosphate (PLA-bioCaP) composite scaffolds in rabbit femoral condyle critical defects. We explored two distinct scaffold designs to assess their influence on bone healing and scaffold performance. Structures with alternate (0°/90°) and helical (0°/45°/90°/135°/180°) laydown patterns were manufactured with a 3D printer using a fused deposition modeling technique. The scaffolds were meticulously characterized for pore size, strut thickness, porosity, pore accessibility, and mechanical properties. The in vivo efficacy of these scaffolds was evaluated using a femoral condyle critical defect model in eight skeletally mature New Zealand White rabbits. Then, the results were analyzed micro-tomographically, histologically, and histomorphometrically. Our findings indicate that both scaffold architectures are biocompatible and support bone formation. The helical scaffolds, characterized by larger pore sizes and higher porosity, demonstrated significantly greater bone regeneration than the alternate structures. However, their lower mechanical strength presented limitations for use in load-bearing sites.

## 1. Introduction

Trauma, systemic diseases, neoplastic fractures, infections, or a compromised blood supply are some of the causes that can result in large bone defects, which can delay or impair healing, leading to a loss of function in the affected individual. Consequently, the need for new treatments for patients with musculoskeletal diseases has increased in recent decades [1,2]. The regenerative capacity of bone has provided a paradigm for developing new bone regeneration strategies, making bone grafting the main treatment for bone injuries [1]. Bone tissue engineering is the most developed research field in tissue engineering. It aims to induce the formation of new functional bone tissues based on the understanding of bone biology and its development [2,3,4].

Large-sized bone defects cannot be healed by themselves when exceeding the so-called critical defect size. Furthermore, different factors may be involved in the failure of said process, such as the patient’s age and gender, unstable biomechanical properties, or an unfavorable wound environment [5,6,7]. In those situations, surgical therapeutic interventions can be required due to the limited intrinsic regeneration potential. In this area, tissue engineering has emerged as a promising approach to bone healing [8].

The search for new bone graft substitutes to face the drawbacks of autografts, allografts, and xenografts [5,9,10] led researchers to explore new combinations of cells, biomaterials, and biological factors to achieve new therapeutic strategies for bone regeneration [9,10,11]. However, none of the currently available bone graft substitutes possess all the desirable biological requirements for a biomaterial, such as bioactivity, biomimetic (including biocompatibility associated with osteoconductive and osteoinductive properties), angiogenic potential, biological safety, low patient morbidity, volumetric stability, biomechanical properties, non-immunogenic, biodegradability, easy market availability, long shelf life, and reasonable production cost [2,10,12].

Among the major bone tissue engineering approaches, novel scaffold-based treatments have recently been widely used [8]. These methods are based on using three-dimensional porous structures that can support and actively guide tissue regeneration. [10,12]. Ideally, scaffolds suitable for bone tissue engineering (BTE) should aim to provide a microenvironment that mimics the properties of the native extracellular matrix (ECM) [13], acting as space holders for cell infiltration, attachment, growth, and differentiation that also improves their viability. Additionally, these three-dimensional structures facilitate the transmission of loads to surrounding tissues, instantly providing mechanical support to the defect site after implantation [2,8,13]. Modifying types of fabrication processes, structural features, biomaterial composition, and biological requirements may modulate these characteristics. The latter is closely related to biomaterial selection and incorporating different substances such as growth factors, stem cells, etc. [2].

Several authors have previously analyzed the importance of different scaffold printing techniques, materials, and designs, such as Garot et al. [12], Amini et al. [3], Ostrowska et al. [14], Liang et al. [15], and Gleadall et al. [16], who demonstrated the importance of scaffolds’ structural geometry.

The design and fabrication of scaffolds were limited in the past since traditional technologies could not incorporate internal architecture and control porosity [12,17,18,19,20]. However, currently, additive manufacturing (AM), also called rapid prototyping (RP) or solid freeform (SFF), has addressed these problems, allowing complete control over scaffold architecture at a very reasonable cost. AM offers the possibility of customizing the scaffold’s global shape and internal structure at high reproducibility and reliability [2,21,22,23]. Additionally, it may be used in personalized medicine since three-dimensional images acquired by Computed Tomography (CT) or Magnetic Resonance Imaging (MRI) can be reproduced layer by layer based on CAD models [12,24,25,26].

Extrusion-based, powder-based, and vat photopolymerization AM techniques allow the manufacture of polymeric, ceramic, and metallic scaffolds. Although AM techniques have been widely used in biomedical research, only a few products are currently available on the market [12]. The desired traits of printable materials for tissue engineering are printability, biocompatibility, and good mechanical and structural properties. Polymers are particularly well suited for the additive manufacturing of scaffolds, and various printing techniques have been employed to fabricate polymeric scaffolds, such as fused deposition modeling, selective laser sintering, inkjet 3D printing, stereolithography, or 3D plotting [24,25].

Polylactic acid (PLA) is one of the most widely used synthetic polymers, commonly used as raw material in the fused deposition modeling-based 3D printing process. Its versatility, mechanical properties, and biodegradability make it an exciting alternative for application in bone tissue engineering. However, PLA also presents several drawbacks, such as slow degradation rate, hydrophobic behavior, and release of acidic byproducts during degradation processes [10,13,25,27,28,29]. To solve polymers’ drawbacks and obtain a more practical, functional, and valuable structure, composite materials are synthesized by combining two or more carefully integrated phases [24,30]. PLA composites can be fabricated by incorporating metal, ceramic, organic, inorganic, or nano-materials into the polymeric matrix [27].

Calcium phosphate (CaP) ceramics, such as hydroxyapatite (HA) or beta-tricalcium phosphate (β-TCP), are some of the most commonly used synthetic bone substitutes due to their composition similarities to natural bone [10,31]. CaP bioceramics are biomimetic materials that stand out for their osteoconductivity, osteoinductivity, biocompatibility, and bioactivity. However, their brittleness represents a major disadvantage, mainly for load-bearing applications [10,12,25]. Hence, the suitability of bioceramics for being printed through additive manufacturing techniques allows the manufacture of composite scaffolds [27,32], which optimizes the biocompatibility of the polymeric materials but maintains adequate mechanical properties and avoids the brittle behavior of the bioceramics [12]. Furthermore, bioceramics also block the acidic environment originating from PLA’s degradation and increase its hydrophilicity and degradation rate [33].

Despite the importance of bone graft composition, some specific requirements regarding the design of the scaffolds are also essential for bone tissue engineering. However, the design and optimization of the scaffolds for successful integration remain unclear, and it has been hypothesized that the results may depend on their fluid flow and nutrient/waste diffusion properties [34,35]. Geometrical characteristics of 3D scaffolds, such as pore architecture, pore size, porosity, and interconnectivity, may greatly influence bone regeneration capacity [5,10,36]. These properties are essential for osteoblast and mesenchymal cell migration, proliferation, and differentiation. Additionally, pore interconnection is essential for tissue ingrowth in porous material since it allows blood vessel invasion and nutrient supply [37]. The scaffolds should also provide an effective support effect and maintain an appropriate mechanical environment at the defect site [38]. Mechanical properties depend on porosity, pore size and shape, and material properties so those characteristics will determine the amount of mechanical stimulus the scaffold carries [35]. All these parameters may be affected by the scaffold’s design, which influences various factors in tissue engineering. Different geometries can be achieved by varying the position and orientation of the fibers, creating structures with aligned or staggered filaments, “repeated layers”, and orientations such as 0°/90° or 0°/60°/120°, depending on the print path [16]. Different authors have already illustrated the effect of varying scaffold designs on bone healing, such as Berner et al. [8], evaluating the effect of scaffold architecture on cranial bone healing; Lim et al. [39], studying the effect of different pore architectures; or Entezari et al. [40], demonstrating that manipulating pore size and permeability of 3D-printed scaffolds is a valuable strategy for enhancing bone regeneration outcomes. However, there is still a gap between the fabrication of different printing paths and their evaluation in vivo in a controlled study, being isolated from any other type of variable that could influence bone growth.

In the present manuscript, 3D-printed composite scaffolds were manufactured using fused deposition modeling to evaluate the impact of different laydown patterns. PLA was chosen as a polymeric matrix to synthesize the scaffolds, and a previously proven shark-teeth marine-derived bioapatite (bioCaP) [36,37,38] was selected as a reinforcing agent. The primary objective of this study is to investigate and compare the effects of two distinct scaffold architectures—alternate and helical laydown patterns—on bone regeneration. This study aims to assess various scaffold properties, including pore size, porosity, mechanical strength, and overall biocompatibility, and their impact on the efficacy of bone healing in critical-sized defects. The null hypothesis was that scaffold architecture significantly influences the rate and quality of bone regeneration in critical-sized defects.

## 2. Materials and Methods

### 2.1. Fabrication of 3D-Printed PLA-bioCaP Scaffold

Previously reported processes [41] were utilized to obtain marine bioderived calcium phosphate (bioCaP) grains from *Prionace glauca* shark teeth as a byproduct provided by IIM-CSIC (Vigo, Spain). The resulting bioCaP particles (20–63 µm diameter) showed a biphasic composition of ~70% apatitic (HA, apatite-CaF, fluorapatite) and ~30% non-apatitic phase (whitlockite, tricalcium bis(orthophosphate)), with contributions of F (1.0 ± 0.5 wt%), Na (0.9 ± 0.2 wt%), and Mg (0.65 ± 0.04 wt%) [41,42].

PLA particles with a diameter of 80–250 µm were obtained from commercial polylactic acid pellets (SMARTFIL^®^, Smart Materials, Jaén, Spain) and then mixed with a contribution of 12.66 wt% of bioCaP using a Turbula^®^ 3D mixer (WAB, Nidderau, Germany). A composite filament was produced using a filament extruder system (Filastruder, Snellville, GA, USA), as published by Rojas-Lozano et al. [43]. This 3D-FDM (fused deposition modeling) printer works with two temperature control points (T1 and T2), whose temperature is adjusted according to the material of the pellet to be extruded. In our case, T1 = 140 °C, T2 = 220 °C.

**Figure 1:**
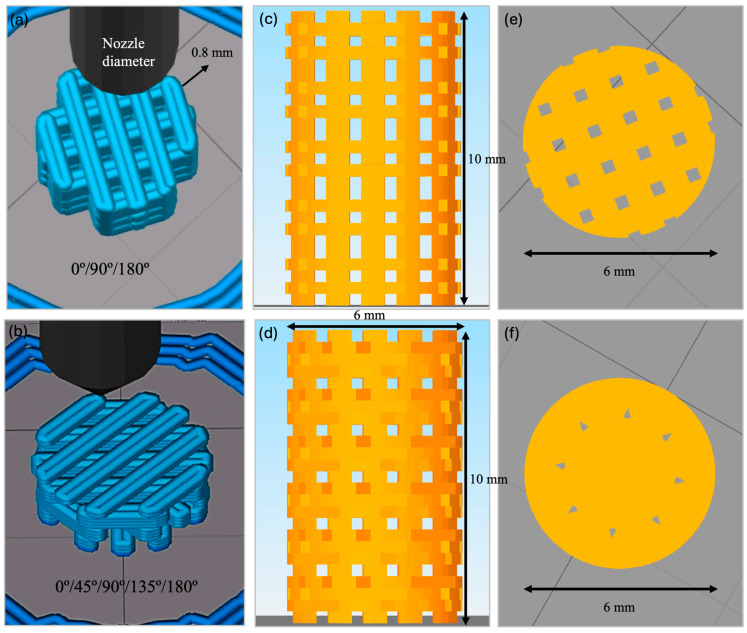
Illustrations from Simplify 3D Professional Software of the 3D printing process of alternate (**a**) and helical (**b**) scaffolds and final open porosity distribution of alternate (**c**,**e**) and helical (**d**,**f**) structures.

The filament was incorporated in a 3D printer (TUMAKER Voladora NX Pellet, Oiartzun, Spain) to manufacture cylindrical scaffolds with dimensions of 6 mm diameter and 10 mm height. A 0.8 mm diameter nozzle was utilized to synthesize scaffolds with two different laydown patterns, namely 0°/90°/180° (alternate structure (ALT)) and 0°/45°/90°/135°/180° (helical structure (HEL)). Simplify3D Professional Software (Version 4.0) was used to show simulations of the printing process and final scaffolds (Figure 1). Before implantation, samples were packed in a laminar flow cabin and sterilized with a 15 kGy dose of gamma radiation.

### 2.2. Characterization of the 3D-Printed Scaffolds

In a previous work, our group performed physicochemical characterization of 3D-printed PLA-12 wt% bioCaP composite scaffolds obtained from the same filament after being subjected to the 3D printer (TUMAKER Voladora NX Pellet, Oiartzun, Spain), as mentioned above. It included the evaluation of the bonding configuration by Raman spectroscopy, with the quantitative analysis of the Raman spectra of different PLA-bioCaP scaffolds with increasing contributions in wt% of bioCaP, to obtain the bioCaP/PLA ratio at the printed scaffolds for each composite filament. Furthermore, the surface topography and the wettability were also, respectively, evaluated by SEM and contact angle measurements. Given that, the present work will focus on the pore morphology, interconnectivity, and mechanical characterization of the mentioned scaffolds, as these data will be of great importance in evaluating the in vivo response of the implanted scaffolds.

#### 2.2.1. Pore Morphology

Three-dimensional images were obtained using Micro-CT (SkyScan 1172, Bruker-microCT, Kontich, Belgium). Scanning parameters were set to 13.54 µm pixels, X-ray sources of 70 kV and 141 mA, and a 0.5 mm Aluminum filter. The samples were rotated 360 degrees around their vertical axis with a rotational step of 0.4 degrees. The raw images of the scaffolds were reconstructed using the Skyscan standard software (NRecon, Bruker-microCT, Kontich, Belgium) to serial coronal-oriented tomograms using a modified back-projection algorithm [44] and subsequently analyzed with another program (CTAn, Bruker-microCT, Kontich, Belgium). For 3D analysis, the volume of interest (VOI) was defined as a cylindrical region of 5.5 mm centered on the scaffold, with a total length of 8.0 mm (591 slides). Then, Anisotropic Diffusion filtering, the histogram-based manual thresholding method [45], and the despeckle tool were applied to the images before quantification of specific parameters such as open porosity, object surface per volume ratio (ObjS/V ratio), strut diameter (defined as strut thickness, St.Th) and pore size (defined as strut separation, St.Sp.). Likewise, interconnectivity was calculated with an algorithm considering only the open and accessible porosity volume within the scaffold, as previously described [46,47].

#### 2.2.2. Mechanical Test

The mechanical behavior of the two different laydown patterns of 3D-printed scaffolds was assessed through compressive mechanical testing (Zwicki, Zwick/Roell, Ulm, Germany). Compression tests were performed, starting with a preloading force set at 0.5 N and then using a load cell of 1 kN. The cylindrical scaffolds were compressed at a 1 mm/min compression speed until failure. The force and displacement were recorded throughout the compression and converted to stress and strain based on the initial scaffold dimensions. The compressive strength was measured at the end of the elastic modulus.

### 2.3. Animal Model

The present manuscript was written following the Animals in Research Reporting In Vivo Experiments (ARRIVE) guidelines [48].

Rabbits are the most used preclinical model for bone tissue testing. In addition to their easy housing and handling, they reach skeletal maturity at an early age after puberty, present similarities in bone mineral density and fracture toughness with humans, and their bone turnover is faster than other species like primates or rodents. Likewise, femoral condyles support defects more significant than 3 mm to test biocompatibility and osteoinduction in cancellous bone, so they allow the performance of critical defects to test biomaterials in load-bearing conditions, which should be 6 mm according to the species and location [6,49,50,51,52]. Critical-sized defects are defined as the smallest wound that does not heal spontaneously over a long period of time, so they are commonly performed to evaluate the scaffold’s bone healing properties [6,33].

Eight healthy adult, skeletally mature male New Zealand White rabbits (4–5 kg, male) were obtained from Granja San Bernardo, Navarra, Spain. This study was conducted in accordance with the Declaration of Helsinki and approved by the Ethics Committee of the University of Santiago de Compostela University (Spain) (Reference Number—02/20/LU-002) and authorized by the Regional Government of Galicia. The animal housing and experimental procedures were conducted in the Animal Experimentation Facility of the University of Santiago de Compostela (Lugo, Spain).

**Figure 2:**
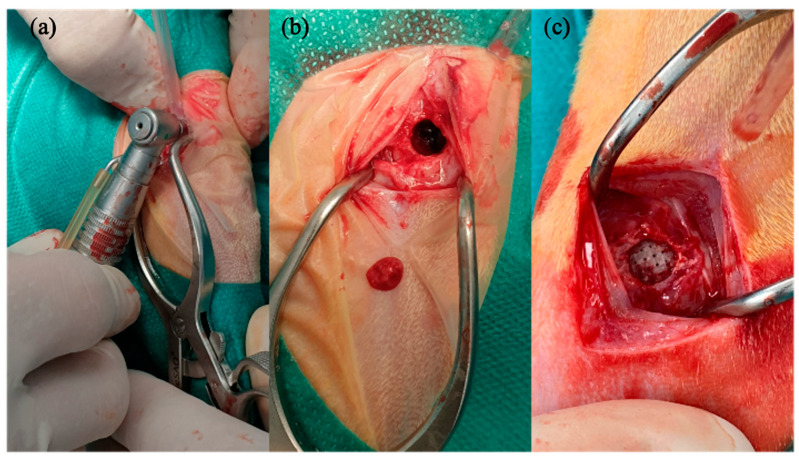
Intraoperative image of defects made in the lateral femoral condyle. Defect performed with a trephine burr (**a**), image after bone fragment dislocation, (**b**) and implantation of the scaffold (**c**).

To perform the surgical procedures, rabbits were premedicated by administering an intramuscular combination of medetomidine (50 ug/kg, Domtor, Esteve, Barcelona, Spain), ketamine (25 mg/kg, Imalgène 1000, Merial, Toulouse, France), and buprenorphine (0.03 mg/kg, Buprex, RB Pharmaceuticals, Berkshire, UK). Then, inhalatory anesthesia (Isoflurane, inspiratory Fraction ISO 2.5–4%, Isova-vet, Schering-Plow, Madrid, Spain) was utilized to induce and maintain general anesthesia. Furthermore, enrofloxacin (5 mg/kg SC, Ganadexil 5%, Invesa, Barcelona, Spain) and meloxicam (0.2 mg/kg SC, Metacam, Boehringer Ingelheim España, Barcelona, Spain) were administrated in order to obtain antibiotic prophylaxis and pain control, respectively. A circular bone defect 6 mm in diameter was performed on the rabbit’s lateral femoral condyle bilaterally after cutting skin and muscle layer by layer, using a trephine burr (227B.204.060, Komet, Germany) connected to a surgical motor under irrigation (Figure 2). The fabricated scaffolds were implanted in the created bone tunnels, and each one was allocated to one of the two treatment groups according to block randomization: PLA-12CaP-ALT (alternate structure) and PLA-12CaP-HEL (helical structure). After suturing, an intramuscular injection of atipamezole (25 µg/kg IM, Nosedorm, Karizoo, Barcelona, Spain) was administered to revert the sedation, and then the rabbits were placed in the cages. Enrofloxacin (5 mg/kg, Ganadexil 10%, Invesa, Barcelona, Spain) and meloxicam (0.1 mg/kg, Metacam, Boehringer Ingelheim España, Barcelona, Spain) were utilized postoperatively for 5 days, with the same aim as reported above. In addition, veterinarians monitored the rabbits weekly for wound dehiscence, inflammation, infection, lameness, and general health.

Twelve weeks later, the animals were sedated with medetomidine (50 ug/kg IM, Domtor, Esteve, Barcelona, Spain) and ketamine (25 mg/kg IM, Imalgène 1000, Merial, Toulouse, France) and then euthanized by sodium pentobarbital (100 mg/kg IV, Dolethal, Vétoquinol, Madrid, Spain) overdose injection in the lateral auricular vein. Samples were dissected free of skin and soft tissue, and femoral condyles were extracted, harvested, and fixed in a 10% buffered formalin solution for 2 weeks.

### 2.4. Micro-CT Analysis

After the sample’s fixation, specimens were scanned using a high-resolution micro-computed tomography (uCT) machine (Skyscan 1172, Bruker microCT NV, Kontich, Belgium) equipped with an 11-Mpixel CCD camera. The acquisition parameters were set as described in a previous section. The reconstruction of the X-ray projections was performed using a modified back-projection algorithm [44] (NRecon v.1.7.5, Bruker, Kontich, Belgium) with a final voxel size of 13.58 µm. The bone regeneration capacity of the implants was assessed using a CT Analyser (CTAn 1.20.3.0+, Bruker, Kontich, Belgium), and parameters such as Bone Volume/Tissue Volume (BV/TV), Bone Surface/Bone Volume (BS/BV), Trabecular Thickness (Tb.Th.), and Trabecular Pattern Factor (Tb.Pf.) were evaluated inside a cylindrical region with a diameter of 5.987 mm and a total height of 6 mm (442 slices), defined as the volume of interest (VOI).

### 2.5. Histologic and Histomorphometric Analysis

The specimens were later processed for undecalcified ground sections according to the method described by Donath [53]. Briefly, the samples were dehydrated with EtOH and embedded within a methylmethacrylate resin (Technovit 7200-VLC, Heraeus Kulzer GmbH, Wertheim, Germany). Resin blocks were cut using a band saw for the purpose of obtaining two central sections from each implant, which were micropolished until they had a thickness of ~40 μm. Finally, tissue slides were stained with Lévai–Laczkó’s protocol.

Once obtained, slides were imaged with an Olympus BX51 microscope (Olympus Corporation, Tokyo, Japan). The images of the whole section were captured at ×4 augments and colored with Adobe Photoshop CS6 (Adobe Systems Incorporated, San Jose, CA, USA), distinguishing new bone tissue, composite material, pristine bone, and soft tissue. Colored images were analyzed using the Olympus CellSens 1.5 (Olympus Corporation) program in order to measure the following histomorphometric parameters: bone-to-implant contact (BIC), Implant Surface/Tissue Surface (IS/TS), and Bone Surface/Tissue Surface (BS/TS) inside a defined Region of Interest (ROI). Scaffolds incorrectly placed proximally in the medullar cavity instead of the trabecular bone were excluded from the analysis.

### 2.6. Statistical Analysis

Data were expressed as means ± standard deviations (SDs). The statistical analysis was performed with SigmaPlot 12.5 software for Windows (Systat Software Inc., Chicago, IL, USA). In the statistical analysis for pore morphology and the mechanical test, the normality of the variables was assessed using the Kolmogorov–Smirnov test or Shapiro–Wilk test, and statistical comparison of different groups’ results was performed through a Paired t-test. Correlation studies were performed using Pearson’s correlation analysis. However, when analyzing micro-tomographic and histomorphometric results, the normality of the variables was evaluated using the Shapiro–Wilk test. Then, the equality of variances was checked through an Equal Variance Test. If both tests were passed, Student’s t-test was used to perform a statistical comparison of the samples belonging to both groups. Nevertheless, a Mann–Whitney U test was selected for normal but non-equal variance variables to compare the results statistically. The statistical significance level was set at *p* < 0.05 for all parameters.

## 3. Results

BioCaP was successfully composited in the PLA matrix for the synthesized scaffolds with different laydown patterns through the fused deposition modeling technique. As mentioned before, the scaffold’s design is a key factor for bone tissue engineering. The following results try to describe how the laydown pattern may affect varying implant characteristics.

**Figure 3:**
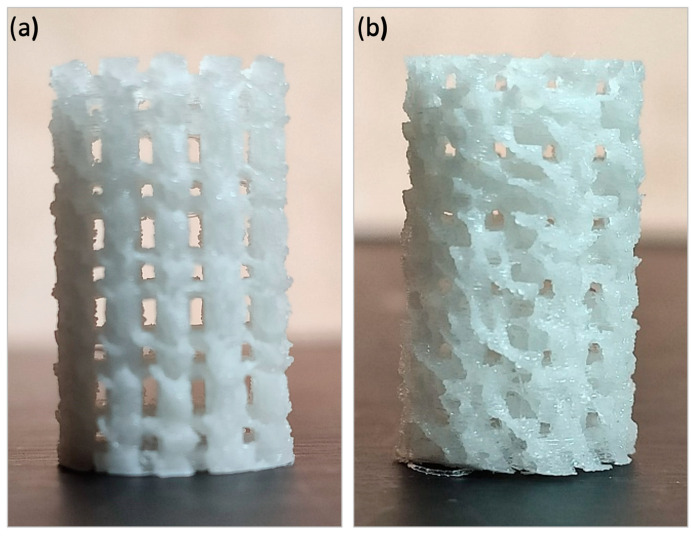
The macroscopic architecture of the 3D-printed scaffolds (Ø: 6 mm × h: 10 mm): alternate structure (**a**) and helical structure (**b**).

Different laydown patterns of the 3D printed scaffold could be observed macroscopically (Figure 3); both structures seemed to be highly interconnected along their circumferences, this being more significant for the helical one. The alternate structure (Figure 3a) showed parallel lines of 0.8 mm thickness with a separation between them of 0.4 mm perpendicularly alternating the direction of the processing lines between each overlapped layer. The helical structure (Figure 3b) also exhibited 0.8 mm thick lines with a separation of 0.4 mm. However, their direction varied in 45 degrees about the already printed lines. This difference resulted in a higher separation among perpendicular struts and a higher pore size. In the case of the alternate structure, regular square-shaped pores could be seen from dorsally and lateral views. Nevertheless, pores in the helical structure presented bigger and more irregular-shaped pores from the lateral and dorsal view (Figure 3).

### 3.1. Pore Morphology

**Figure 4:**
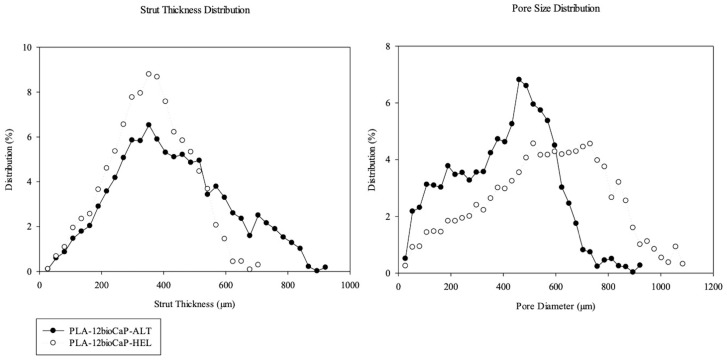
Strut thickness and pore size distribution of 3D-printed composite scaffolds.

Three-dimensional micro-tomographic images confirmed the pore size and shape differences. Furthermore, 3D analysis was performed to quantify them (Table 1). Firstly, printing patterns had great importance in the degree of infilling of the scaffolds, so this explained why alternate structures, with a higher infill ratio, presented higher scaffold volumes than helical ones (100 ± 10 mm^3^ vs. 70 ± 2 mm^3^). Furthermore, the object surface per volume ratio (Obj.S/V) was evaluated and the obtained information suggested that helicoidal structures provided more surface area available for biological contact than the alternate groups, with mean values of 10.6 ± 0.7 mm^−1^ and 8 ± 2 mm^−1^. The porosity of the scaffolds was evaluated in samples with alternate and helical structures. The results confirmed what was suspected, obtaining mean porosity values of 45 ± 6% and 63 ± 1%, respectively. Moreover, the pore size was determined, with average values recorded at 400 ± 20 μm for alternate structures, and 560 ± 6 μm for helical formations. Regarding pore size distribution (Figure 4), a more comprehensive range of pore sizes could be observed for helical structures, with peaks at 514.8 μm and 731.5 μm. However, the range was narrower in the case of alternate structures, with smaller pore sizes and a high peak at 460.6 μm. Thus, this issue provides the helical structure with better features to promote bone cell growth, neovascularization, and the diffusion of nutrients, oxygen, and waste products.

**Figure 5:**
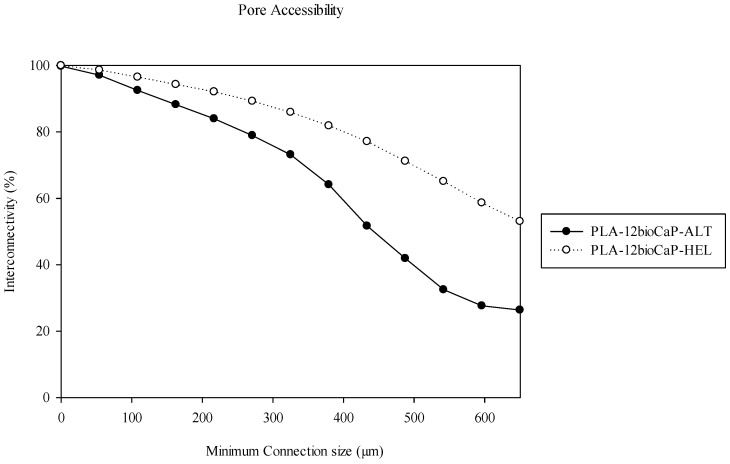
Pore accessibility assessment in micro-CT.

Additionally, to confirm this statement, a detailed study of the interconnectivity was performed through the pore accessibility or open and accessible porosity volume within the scaffold. This method gives the smallest pore constriction connecting every voxel of the scaffold, quantifying bone ingrowth as a function of accessible pore size [54]. So, if the interconnective pore size is 0 (m), the effective interconnectivity will be 100% since all the pores will be counted in the porosity [47]. Although both structures were highly interconnected, the analysis showed notable differences. As can be seen in Figure 5, interconnectivity values lower than 90% were achieved at minimum connection sizes of 162.6 μm for the alternate structure and 270.9 μm for the helical structure. Likewise, the difference between them increased as the percentage of interconnectivity decreased, reaching values less than 80% when openings were greater than 270.9 μm in the alternate group and 433.5 μm in the helical group. The highest pore throat studied was 650.2 μm, providing pore accessibility percentages of 26% and 53% for both structures. 

Concerning strut thickness, higher mean values were appreciated in the alternate group compared with the helicoidal one (430 ± 80 μm vs. 350 ± 20 μm) (Table 1). Furthermore, apparent differences could also be observed between alternate and helical structures regarding strut thickness distribution (Figure 4). Even though both structures peaked at 352.2 μm, the alternate one presented a more comprehensive range of sizes, reaching strut thicknesses up to 800 μm. The differences between laydown patterns probably caused these results since the filaments were aligned in the alternate structure, and they could merge and be interpreted as one during micro-tomographic analysis. Due to its layer configuration, something that will hardly happen in the helical structure involves the deposition of staggered fibers varying by 45 degrees. 

**Figure 6:**
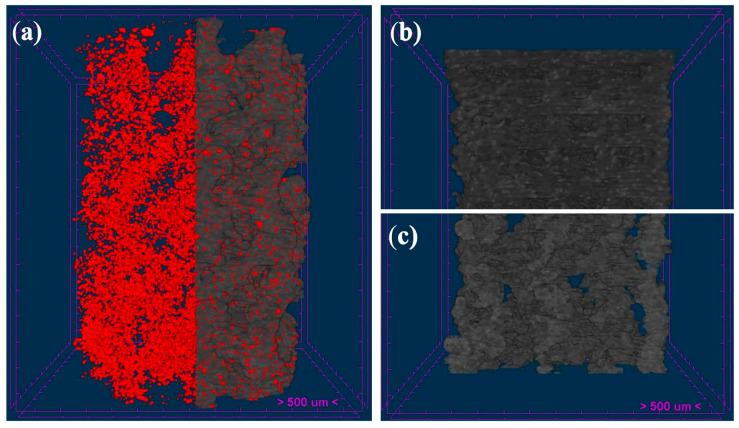
Micro-CT reconstruction of 3D-printed scaffolds. Distribution of bioCaP particles (red dots) inside the struts along the scaffold (**a**), and cross-sections of alternate (**b**) and helical (**c**) structures.

The micro-tomographic evaluation also allowed us to confirm an adequate dispersion of the bioCaP particles along the longitudinal and cross-sectional axes of cylindrical structures. Figure 6 shows the homogeneous dispersion of the particles in the surface and the inner part of the struts.

### 3.2. Mechanical Test

**Figure 7:**
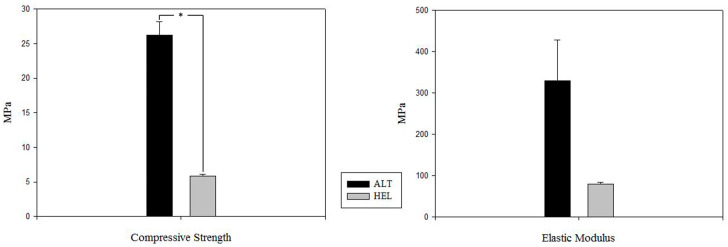
Results of mechanical tests. All parameters are represented as mean ± SD (* *p* < 0.05). MPa: megapascal, ALT: alternate structure, HEL: helical structure.

The implications of the laydown patterns in the compressive strength were also evaluated previously for scaffold implantation due to their great importance when facing bone regeneration in load-bearing sites. Four alternate and helical group samples were evaluated, and calculated strength was obtained from the end of the linear elasticity phase. The results are visible in Figure 7, showing a marked difference in compressive strength and elastic modulus values between both structures, which proved to be statistically significant. Therefore, the alternate structure will be significantly more resistant than the helical one.

In addition, the correlation between the compressive strength and several micro-tomographic parameters, such as scaffold volume, porosity, and strut thickness, was assessed. As demonstrated in Table 1, printed laydown patterns resulted in high differences in scaffold volume, and a positive correlation between these data and compressive strength was confirmed by Pearson’s correlation method. Furthermore, a negative correlation with this parameter was found for the porosity but not the strut thickness (Table 2). Consequently, the weakness of the helicoidal structure could be closely related to its lower volume and increased porosity. 

### 3.3. Animal Model

In vivo trials were performed without complications, and all the animals recovered adequately after the surgery. Furthermore, the skin healed normally, and no signs of infection, inflammation, or dehiscence were found. During the postoperative period, one of the rabbits presented a marked lameness of the left hind limb, although it could use the leg without problems. Despite being treated with analgesic drugs, it maintained a slight lameness until the time of euthanasia. 

Macroscopically, mild to moderate signs of osteoarthritis, secondary to the surgical process, were observed in almost all the rabbits. Several related findings were described, such as thickening of the joint capsule, excessive synovial fluid, and loss of articular cartilage. During the necropsy, total and partial fractures of the lateral femoral condyle were also detected in two knees; therefore, they were excluded from the analysis. Additionally, macroscopical examinations confirmed that bone defects healed successfully, without signs of bone hematoma or infection being present, and the scaffolds seemed to be adequately anchored to the trabecular bone of the femoral condyle.

An in-depth analysis of these findings could demonstrate that both fractured femoral condyles were related to implanting scaffolds with a helical structure. The low mechanical resistance of the implants could lead to a collapse of the structure and a subsequent fracture of the bone when implanted in load-bearing sites. 

### 3.4. Micro-CT

**Figure 8:**
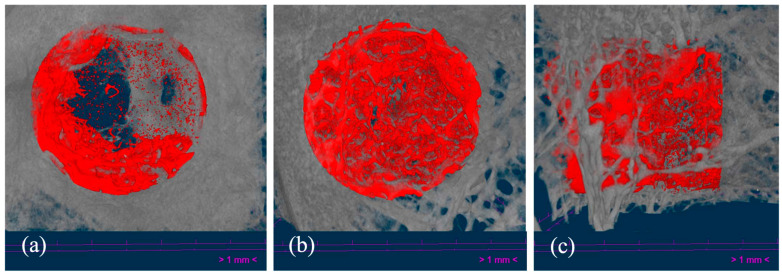
Representative micro-CT images of the implanted alternate (**a**) and helical scaffolds, in coronal (**b**) and sagittal (**c**) views, 12 weeks after the implantation. The measured volume of interest (VOI) was highlighted in red color.

**Figure 9:**
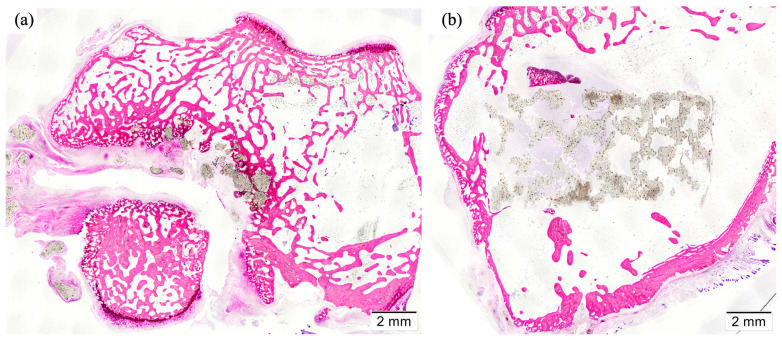
Histological sections of the samples were excluded from the analysis. Fractured femoral condyle and breakage of the scaffold (**a**) and scaffold implanted inside the bone marrow (**b**).

Three-dimensional reconstructed images obtained by micro-CT showed appropriate osseointegration of the implants within the femoral condyle, with new bone formation occurring through the scaffolds. Representative images are included in Figure 8. Generally, no striking signs of inflammation or rejection were observed, with trabecular bone ingrowth inside the scaffold pores. Furthermore, three-dimensional views helped to clearly distinguish those samples where the femoral condyle and the scaffold were fractured (n = 2) and those in which the implantation was performed in a more proximal femoral site and the scaffold was located inside the bone marrow and not in the trabecular bone (n = 1). These findings were also confirmed histologically, and both were considered criteria for excluding the analysis of the respective samples (Figure 9). 

**Figure 10:**
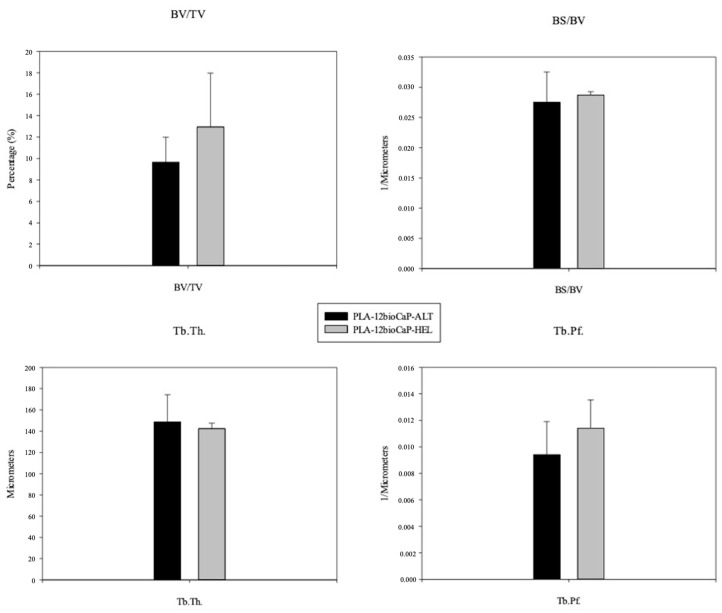
Micro-CT histomorphometric measurements. BV/TV: Bone Volume/Tissue Volume, BS/BV: Bone Surface/Bone Volume, Tb.Th.: Trabecular Thickness, and Tb.Pf.: Trabecular Pattern Factor.

Bone regeneration inside the VOI was evaluated through a micro-tomographic analysis, and values regarding the following histomorphometric parameters were obtained: BV/TV, BS/BV, Tb.Th., and Tb.Pf. Significant differences between alternate and helical structures for any of the analyzed parameters were not found. As shown in Figure 10, the amount of newly formed bone was higher for the helical structure than the alternate structure, with BV/TV percentages of 12.94 ± 5.0% and 9.65 ± 2.3%, respectively, and likewise for BS/BV and Tb.Pf. There were slight variations among structures, indicating that the available bone surface and the intertrabecular connectivity ratio were mildly higher in helical structures. However, regarding trabecular thickness, newly formed bone trabeculae seemed slightly thicker for alternate structures.

### 3.5. Histology

**Figure 11:**
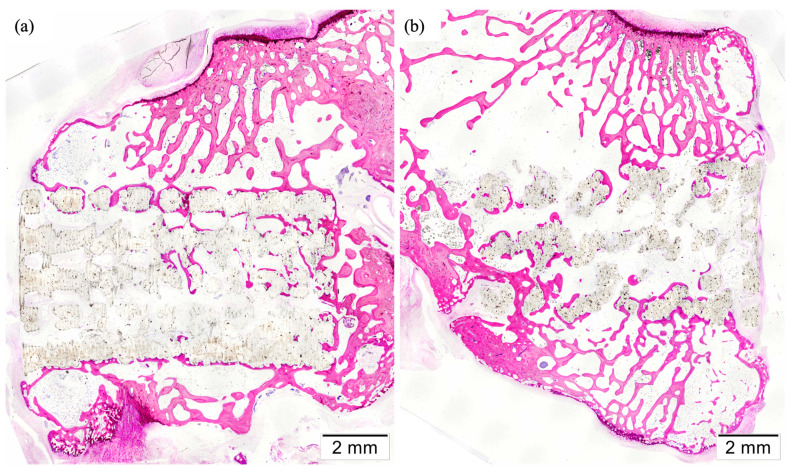
Histological sections showing the effect of 0°/90°/180° (**a**) and 0°/45°/90°/135°/180° (**b**) laydown patterns on bone regeneration 12 weeks after the implantation of the scaffolds in femoral condyle defects.

The effect on bone regeneration was evaluated by creating bone-critical defects in a rabbit femoral condyle model. After twelve weeks of implantation, specimens were processed, and two slides were obtained from the central sections of each one. Then, they were stained with Lévai–Lazckó’s protocol and evaluated histologically by optical microscopy. Representative histological images are included in Figure 11.

All the implants maintained their position in the center of the defect and successfully osseointegrated into the host bone, showing good biocompatibility. The absence of fractures or displacements of the grafting materials was described, except for those specimens in which a total or partial lateral condylar fracture occurred (Figure 9). Most of the bone-to-implant contacts were detected in the periphery of the implants, where their surfaces were exposed and in contact with the trabecular condylar bone. In these sites, bone ingrowth could be appreciated through the pores to the inner parts of the scaffolds, more notably in those with a helical structure, probably due to its higher porosity and available surface. However, non-major differences were observed subjectively regarding the regenerative potential of both structures, which presented similar interconnected trabecular systems. The quantification of the newly formed bone, the amount of remaining composite material, and the percentage of bone-to-implant contact were performed objectively in the following section.

Likewise, in those sites where the scaffolds were in contact with bone marrow or connective tissue, the pores showed an abundant infiltration with fatty and fibrous tissue. Neovascularization was described inside the pores and mixed with both kinds of tissues. Generally, inflammatory cells were mainly observed surrounding the composite material, specifically macrophages, which were more numerous when compared to slides from the helical group. Their presence could be related to the degradation of resorbable biomaterials, and it seemed clear that the helical structure suffered a higher degree of degradation than the alternate one. Additionally, small infiltrates of lymphoplasmacytic cells were found embedded inside the connective tissue, and some neutrophils were occasionally observed. However, no signs suggested the presence of infection, tissue necrosis, or rejection.

**Figure 12:**
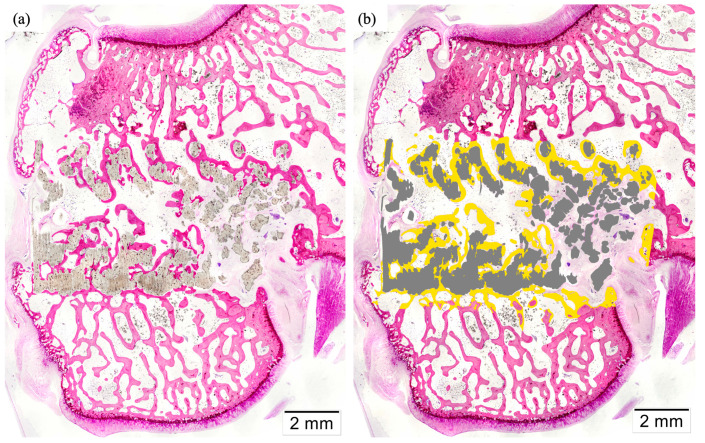
Histological Lévai–Laczkó stained non-colored (**a**) and colored (**b**) sections of helical scaffolds implanted in rabbit femoral condyle.

**Figure 13:**
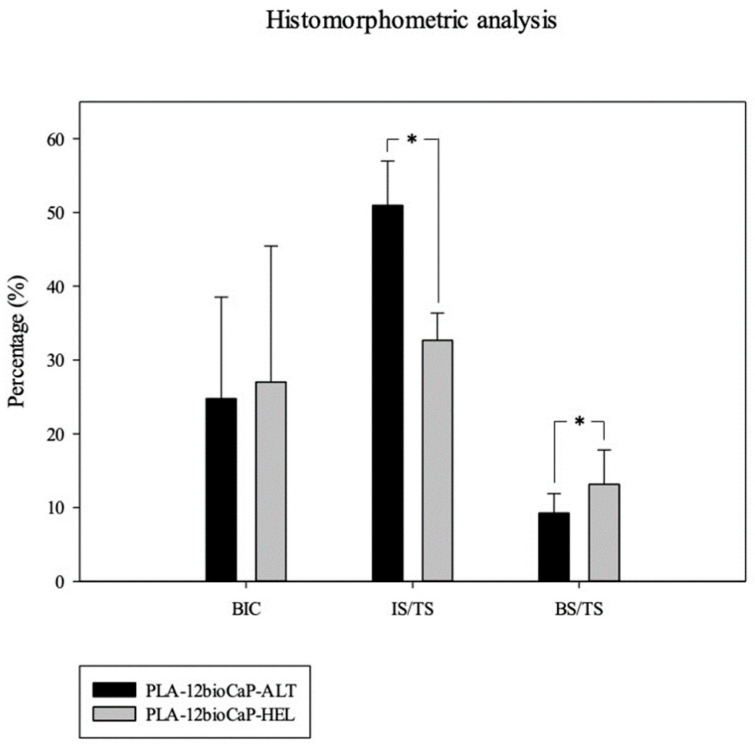
Histomorphometric measurements. BIC: Bone-to-implant Contact, IS/TS: Implant Surface to Tissue Surface, BS/TS: New Bone Surface to Tissue Surface. * *p* < 0.05.

Histomorphometric measurements were performed after coloring the images in a defined Region of Interest, delimited by the trephine burr’s performed defect (Figure 12 and Figure 13). In the images, these limits are represented as well-defined cuts in the lamellar bone perpendicular to the cortical bone. The analyzed parameters were introduced previously, and the following results were obtained. Bone-to-implant contact (BIC) notably, as already described, mainly affected the outer section of the cylinders. The percentage of BIC was similar for both studied types, without significant differences between them. Mildly lower values were found for the alternate structure (24.783 ± 13.741%) compared to the helical one (27.037 ± 18.439%). Then, the amount of biomaterial inside the ROI, defined as Implant Surface to Tissue Surface (IS/TS), was measured. Statistically significant differences were found among structures for this parameter, and mean values were set at 50.950 ± 6.023% for alternate scaffolds and 32.683 ± 3.668% for helical scaffolds. These differences could be explained mainly by the discrepancies between both groups regarding the scaffold’s volume. 

Nevertheless, the biodegradability of the implants should also be considered since a higher available surface for scaffold–environment contact will lead to further degradation, decreasing the IS/TS obtained values. Finally, one of the most important parameters for evaluating the osteogenic potential of the different structures is the Bone Tissue-to-Tissue Surface ratio (BS/TS). Histomorphometric analysis revealed statistically significant differences between the helical and alternate structures when measuring the newly formed bone (BS/TS). The implanted scaffolds with a helical disposition achieved BS/TS mean values of 13.13 ± 4.70%, significantly higher than the ones obtained by the alternate structures (9.246 ± 2.64%). Thus, the characteristics of the helical structures seemed to be more adequate to achieve a more significant osteogenic potential for bone regeneration in critical defects.

## 4. Discussion

Three-dimensionally printed scaffolds with different laydown patterns were designed and fabricated in the present manuscript to assess their morphological differences through the scaffold’s characterization and their capacity to promote bone regeneration when implanted in critical-sized bone defects. The results showed that helical structures presented higher values of pore size, porosity, and pore accessibility compared to the results of newly formed bone.

Most published manuscripts are based on searching for the material or the combination of several that provide optimal conditions for bone regeneration [28,29,30,55,56]. During recent decades, the tissue engineering field has evolved from particulate materials to more complex structures called scaffolds, which provide mechanical support and promote cell growth and vascularization. Furthermore, custom-designed 3D printing has elevated these structures to another dimension, allowing complete control over the geometry of the implants [39,57,58]. Thus, in addition to material selection, the scaffolds’ design has been postulated as another key aspect when facing bone tissue engineering. Understanding how the architectural properties work gives a better insight into the optimal structural design to improve bone regeneration could be provided [40]. Currently, methods such as layer-by-layer deposition (additive manufacturing) are widely used to design complex porous scaffolds with well-defined architecture and optimized pore interconnectivity [3].

Shark teeth-derived bioapatites (bioCaPs) have been studied as an alternative bioceramic material for bone regeneration, which were obtained as fishing byproducts of *Isurus oxyrinchus* and *Prionace glauca* [41,59]. Their biocompatibility has been proved in vitro [59] and in vivo [41], also demonstrating their osteointegrative, osteoconductive, and osteoinductive properties. This bioceramic is based on a globular porous morphology with a biphasic composition of ~70% apatitic (HA, apatite-CaF, fluorapatite) and ~30% non-apatitic phase (whitlockite, tricalcium bis(orthophosphate)) together with contributions of F (1.0 ± 0.5 wt%), Na (0.9 ± 0.2 wt%), and Mg (0.65 ± 0.04 wt%). The presence of these ions contributes to bone healing and regeneration since F enhances the synthesis of bone cell growth factor, and Mg is involved in synthesizing the parathyroid hormone that regulates bone homeostasis [59]. This study confirmed the suitability of this biomaterial to be composited with a widely studied polymer, such as PLA [28,29].

The structural geometry or design of the scaffold is determined by the position and orientation of the fibers, affecting parameters such as pore size, porosity, mechanical properties, and biological performance [16]. The pore features of the 3D-printed scaffolds play an essential role in cell adhesion, proliferation, and migration [60]. As mentioned, the scaffold should be a 3D network with highly interconnected pores. Macroporosity promotes cell and ion transport and, consequently, osteogenesis, and microporosity (<10 μm) improves the surface area and roughness, providing attachment points for osteoblasts [61,62]. Currently, no consensus on the optimal pore size has been achieved, and approaches mainly include regular and irregular pore structures [38,60]. Mean pore sizes ranging from 50 to 900 μm have been used in bone tissue engineering. The minimum recommended pore size is 100 μm; however, sizes under 300 µm still limit angiogenesis, resulting in small blood vessel diameter and bone formation due to reduced oxygen and nutrient diffusion. Larger ones provide more space for cell migration, tissue ingrowth, and vascularization, increasing osteoblast proliferation and differentiation throughout the scaffold [3,60,61]. In addition, porosity and connectivity are critical parameters closely related to pore sizes. High porosity and large pores are supposed to enhance bone ingrowth and osseointegration [3,63,64,65]. Trabecular bone is characterized by a highly trabecular foam-like cellular microstructure, with porosity levels ranging from 30 to 95%, thus establishing ideal values [60,66]. Despite some reports showing differences in osteogenic income of implants with different porosities, no beneficial effects of low porosities were reported either [62]. Other features, such as the materials’ degradation rate and mechanical properties, should be considered when assessing porosity. Those materials with high degradation rates should not have high porosities (>90%) because higher surface areas interacting with host tissue will accelerate degradation due to macrophages via oxidation and/or hydrolysis. However, those with low degradation rates and robust mechanical properties can be highly porous [62]. 

Alternate and helical structures resulted, respectively, in mean pore sizes of 400 ± 20 μm and 560 ± 6 μm, and porosities of 45 ± 6% and 63 ± 1%. After being implanted, helical PLA/bioCaP composite scaffolds achieved higher values of newly formed bone than alternate ones. Greater pore size and the large porosity of the helical structure and a greater available surface area could explain the differences in bone regeneration between both groups, according to what was stated above. The effect on tissue formation of different 3D-printed scaffold designs has been extensively studied [19,31,35]. However, no similar studies in vivo with PLA composites were found. 

Nevertheless, larger pore sizes do not always mean a higher percentage of bone regeneration, mainly when working with sizes greater than 800 μm. Liu et al. [60] manufactured macropore-sized (800, 1200, and 1600 μm) bioceramic (biphasic calcium phosphate, BCP) scaffolds with an identical porosity of 70%, and in vivo trials showed higher BV values for the BCP 800 and 1200 groups than for BCP 1600. This was explained because the surface area of porous scaffolds is closely related to bone formation, and the specific surface area of the scaffolds decreases with the increasing pore size.

To study the effect of pore size and permeability, Entezari et al. [40] fabricated Strontium-doped (Sr-HT-Gahnite) scaffolds with four different architectures, maintaining the same interconnectivity (100%) and similar porosity (49.3 ± 1.9%). Architecture A was a conventional square mesh-like pattern; Architecture B was a double-lined pattern of bimodal pore sizes; Architecture C was a displaced double-layer pattern; and Architecture D was a quatrefoil pattern. Micro-tomographic results showed that Architectures B and D presented greater volumes of regenerated bone. Likewise, both presented higher permeability and larger pore sizes compared to the other two architectures. The authors concluded that the higher tortuosity induced by displacing the layers (C) limited nutrient transportation and formed pillar-shaped bone constructs to carry bone loads. They also found that pore sizes to enhance bone formation should range between 390 and 590 µm since larger pores did not show any improvements. Those findings agreed with our results. 

Even though our manuscript did not analyze the permeability, an equivalent parameter, such as pore accessibility, was measured. It varies as a function of the scaffolds’ porosity and pore size [40], and it reflects the interconnectivity of the network of pores, giving the smallest pore connection (pore throat) between the scaffold edge and any voxel within the scaffold pore space [54]. Pore throat size determines whether cells can enter the pore structure smoothly and affects cell proliferation and differentiation functions since more pore accessibility provides the cells with a larger surface for attachment [38]. In vivo trials confirmed these statements, showing higher values of bone formation and better access of cells to the inner parts of the scaffolds in the helical structures, with superior pore accessibility. However, based on the results, both structures should ensure mass transfer and oxygen perfusion to allow bone regeneration [16].

Regarding the effect of different laydown patterns, several authors studied their effects in vitro and in vivo. Domingos et al. [67] designed polycaprolactone (PCL) scaffolds, varying the pore size, with filament distances from 550 to 750 µm, and the laydown pattern, changing the deposition angle (0°/90°, 0°/60°/120° and 0°/45°/90°/135°), but maintaining a regular filament distance of 650 µm. Increasing the deposition angle achieved quadrangular, triangular, and complex polygonal internal pore geometries without modifying the scaffolds’ porosity. Then, biological experiments were carried out using hMSCs (human mesenchymal stem cell cultures), and a strong influence of pore size and geometry on cell viability was observed. Briefly, larger pore sizes could accommodate more viable cells, probably related to a large surface area and higher porosity. Regarding the influence of pore shape, a decreasing number of deposition angles resulted in incrementing cell viability, revealing that quadrangular pores (0°/90°) improved cell accessibility and colonization [67]. Similarly, Kook et al. [34] manufactured PCL scaffolds with combinations of different laydown patterns (0°/45° or 0°/90°) and pore sizes (150, 250, and 350 µm). Then, the optimum scaffold architecture was determined by an MMT assay to measure the proliferation of MC3T3-E1 cells. Highest cell proliferation was observed in scaffolds with 0°/45° strut layout pattern and 150 µm pore size; thus, based on these results, the authors selected scaffolds with the parameters of 45°/150 µm and 45°/350 µm for in vivo trials. Their results were contrary to those obtained by Domingos et al. [67], who found that the most suitable laydown pattern in vitro was 0°/90°, but agreed with those obtained in the present manuscript. However, Kook et al. [34] found that after in vivo implantation, the scaffolds with 150 µm pore size achieved higher amounts of newly formed bone than those with 350 µm, which is controversial considering the abovementioned results. The absence of a unanimous agreement on optimal pore size and shape can be attributed to the variability in materials employed and the specific implantation sites [5]. Additionally, the body of research examining scaffold geometry through in vivo studies is comparatively small. Variations in pore shape for bone regeneration were studied by Berner et al. [8], where they developed silanized polycaprolactone/tricalcium phosphate scaffolds with 0°/90° and 0°/60°/120° fiber orientations and implanted them in rat skull defects. The analysis showed that a higher degree of newly formed bone was regenerated in the 0°/90° scaffolds compared to the 0°/60°/120° ones, where bone formation was closer to the host bone with less ingrowth to the center of the scaffold. The authors reported that the differences could not only be explained by the variations in higher porosity, large pore size, and lower surface area of the 0°/90° groups, but also the fact that struts’ architecture must play a main role in bone formation.

In addition, one of the challenges when studying the effects of pore shape is the lack of isolation of architectural parameters. Mirkhalaf et al. [36] synthesized Baghdadite 3D-printed scaffolds with five different structures, maintaining similar values of pore size (500 µm), porosity (50%), and surface-to-volume ratio (10 mm^−1^). They were implanted in rabbit cranial defects to assess the effects of the surface convexity (cylindrical struts with convex surfaces vs. concave surfaces), the relative orientation of the scaffold concerning the defect site (scaffolds with cubic pores vs. rotated cubic pores), and interconnectivity (body-centered cubic scaffold). The results showed that only pore interconnectivity significantly affected the scaffold’s bone tissue regeneration capacity, which agrees with the data obtained for alternate and helical structures, where the one with greater interconnectivity obtained better results in bone regeneration trials.

Another essential requirement for bone regeneration is the degradability of the implants, which, regardless of the material, is closely related to the porosity, the pore size, and the interconnectivity. In the present manuscript, the degradation of the scaffolds was subjectively more evident in the helical group, with lower values of IS/TS and a lack of connection between the struts in the histological sections. The degradation pattern of porous structures is linked to pore size, so smaller pores result in slower hydrolysis and, thus, low degradation rates. As expected, the same happens with the porosity since higher values result in further permeability and faster degradation. Likewise, it was observed that scaffolds with large pore sizes and lower porosity degraded faster than those with smaller pore sizes and higher porosity due to the effect of higher available surfaces in scaffolds with macropores [60,68]. Additionally, scaffolds with square pores provided faster degradability and higher weight loss than other pore morphologies such as triangular or parallelogram [69]. By contrast, Domingos et al. [70] reported after in vitro degradation studies that the degradation rate of PCL scaffolds was notably affected by porosity and pore size. At the same time, 0°/90° and 0°/45°/90°/135° filament orientations resulted in similar degradation rates when porosity and pore size values remained similar. 

The scaffold design also has an essential effect on the mechanical properties of the scaffolds, since as porosity and mean pore size increase, the mechanical strength is sacrificed. Thus, an adequate balance among these parameters is essential for synthesizing an ideal scaffold for bone tissue engineering [3]. The present manuscript shows that helical structures demonstrate much lower mechanical properties than alternate structures. Furthermore, this lack of mechanical resistance in helical scaffolds could be the reason for the fracture of two femoral condyles after the scaffold’s implantation in rabbits, even though their compressive strength values were within the range for those stipulated for natural bone (2–12 MPa) [71]. Similarly, Domingos et al. [67] reported that increases in pore size (filament distance from 550 µm to 750 µm), reductions in laydown patterns’ deposition angle, or increases in the number of deposition angles (from 0°/90° to 0°/45°/90°/135°) resulted in scaffolds with lower compressive modulus, and therefore in weaker structures. The authors’ explanation for these results was the larger fused area between struts occasioned by a reduced deposition angle, leading to lower local stress experienced by scaffolds and a greater ease of sliding, increasing the scaffold’s deformability [67,72,73]. Furthermore, high mechanical properties in scaffolds with 0°/90° and 0°/60°/120° laydown patterns are related to the aligned crossover points on every layer. By contrast, 0°/45°/90°/135° orientations produced misaligned crossover points among different layers, and thus the elastic modulus may be lower [16]. Nevertheless, Liu et al. [60], who synthesized scaffold using a brittle material such as BCP, observed that an increase in the size of the macropores resulted in similar compressive strength values.

Regarding differences between scaffolds with aligned or staggered fibers, many authors confirmed that staggered filaments had notably lower mechanical properties than aligned filaments, as reviewed by Gleadall et al. [16]. Specifically, Serra et al. [74] described that PLA-based composite scaffolds with staggered fibers showed 50–75% lower elastic modulus than others with aligned fibers. Likewise, regarding the mechanism by which scaffolds collapse, it could be appreciated that a solid column from the top to the bottom of the scaffolds, as happened with those with 0°/90° laydown patterns, provides it with a pillar that strongly resists compression. When fibers are staggered, filaments bend slightly, and the structure easily collapses in a concertina manner [16].

The present manuscript delved deeper into how specific scaffold designs resulted in significant changes in pore size, porosity, interconnectivity, available implant surface, and mechanical properties. However, it also demonstrated, through the implantation of the scaffolds in an animal model, the effects of these modifications on bone regeneration, with their subsequent implications in the context of bone tissue engineering. 

This study’s results confirmed the initial hypothesis since they demonstrated that scaffold architecture could influence bone regeneration capabilities. Changes deriving from printing paths, which theoretically should trigger a series of biological changes in vivo, favored or hindered bone ingrowth through different mechanisms mainly by facilitating or not facilitating the access of cells and new vessels to the inner part of the scaffolds, but also because of their differences in degradability rate. This study provides novel and interesting information to a field where significant advances are still necessary, mainly in the applications and evaluation in vivo of the different designs. 

In addition, this report presents several constraints that should be pointed out. First, the limited osteogenic properties of the implants, although this was not one of the study’s main goals, due to the already described drawbacks of PLA and the impossibility in our case of manufacturing scaffolds with bioCaP concentrations higher than 12%, which could explain the low obtained values of the newly formed bone. Additionally, the lack of degradation analysis prevents us from ensuring different degradability rates among both structures and their implication on scaffolds’ mechanical integrity. Regarding the animal model, the low number of animals used and the exclusion of several samples may limit the scope of the study. In the same way, long-term clinical trials could be interesting in assessing the performance of the different scaffold designs over time. Furthermore, the variable distribution and limited amount of trabecular bone in the rabbit’s femoral condyle, together with the use of long scaffolds (1 cm), failed in the task of maximizing the contact surface between host bone and implant, being in contact in some cases largely with bone marrow. So, the use of other animal models could be addressed for future research. 

The publication of controversial results regarding scaffold architecture, as presented in this section, highlighted the importance of performing further investigations on this topic to validate and expand the knowledge. The synthesis and comparison of a wider variety of scaffold laydown patterns could provide more interesting information on physicochemical characterization and degradation analysis. Additionally, the analysis of their osteogenic capabilities in in vivo trials is also crucial since in vitro findings are not always correlated with in vivo ones [12]. New alternative approaches may also be used to search for highly porous and interconnected but, at the same time, resistant structures. An example is bimodal pore topologies, described by Entezari et al. [35], which allowed the creation of larger pores without increasing porosity or sacrificing mechanical properties but enhancing the volume and functionality of newly formed bone. Another alternative is the combination of different laydown patterns, such as 0°/90°/180° and 0°/45°/90°/135°/180° orientations, to achieve porous structures that facilitate bone ingrowth without compromising its mechanical resistance. Likewise, using different biomaterials, such as composites made with copolymers and 3D printing techniques, to fabricate the scaffolds could be interesting since biomaterials’ characteristics influence printing patterns and techniques regarding ductility, printability, and mechanical resistance. 

Challenges in the clinical adoption of 3D-printed scaffolds include the need for medical-grade materials, high fabrication costs for patient-specific products, clinician training, and scaffold sterilization [12]. Research and additive manufacturing efforts must focus on these areas to enhance clinical applicability. Adapting techniques for bone applications to weight-bearing sites, like long bone defects, is complex due to different bone environments and implant requirements. Therefore, extensive preclinical studies are crucial for clinical relevance, particularly on larger animals at intended implantation sites.

## 5. Conclusions

This investigation delves into the impact of scaffold architecture on bone regeneration, explicitly focusing on the performance of alternate and helical laydown pattern scaffolds made of polylactic acid–bioceramic phosphate (PLA-bioCaP) composites. This study, conducted in a rabbit femoral condyle critical defect model, meticulously evaluates these scaffolds’ physical characteristics like pore size, porosity, mechanical strength, and biological efficacy. Our results reveal that both scaffold designs are biocompatible and facilitate bone growth, with the helical scaffolds showing a notable advantage in enhanced bone regeneration due to their larger pore size and increased porosity. However, this architectural benefit comes at the cost of reduced mechanical strength, posing limitations for their application in load-bearing areas. This research underscores the critical role of scaffold architecture in bone tissue engineering and highlights the intricate balance required between mechanical stability and scaffold porosity for optimal bone healing. The insights gained from this study are instrumental for future advancements in scaffold design, particularly for applications involving critical-sized bone defects. It opens avenues for exploring hybrid scaffold designs that synergize the benefits of both architectures, aiming for an optimal balance between structural integrity and regenerative capacity.

## Figures and Tables

**Table 1:** Pore morphology measurements in micro-CT. All parameters are represented as mean ± SD. Obj.S/V: object surface per volume ratio, mm: millimeters, µm: micrometers, ALT: alternate structure, HEL: helical structure.

	Scaffold Volume	Obj.S/V	Open Porosity	Strut Thickness	Pore Size
	mm^3^	mm^−1^	%	μm	μm
ALT	100 ± 10	8 ± 2	45 ± 6	430 ± 80	400 ± 20
HEL	70 ± 2	10.6 ± 0.7	63 ± 1	350 ± 20	560 ± 6

**Table 2:** Correlation study between compressive strength and the scaffold’s architectural characteristics (* *p* < 0.05).

Pearson’s Correlation Coefficient	Scaffold Volume	Porosity	Strut Thickness
Compressive Strength	0.98 *	−0.98 *	−0.85

## Data Availability

Data supporting the reported results can be requested to the authors.
